# Response to Hewitt et al, “Overlap of generalized morphea and eosinophilic fasciitis after recreational exposure to epoxy resin”

**DOI:** 10.1016/j.jdcr.2025.04.037

**Published:** 2025-05-24

**Authors:** Daniel R. Mazori, Kristen I. Lo Sicco, Avery H. LaChance, Ruth Ann Vleugels, Alisa N. Femia

**Affiliations:** a The Ronald O. Perelman Department of Dermatology, NYU Grossman School of Medicine, New York, New York; b Department of Dermatology, Brigham and Women’s Hospital, Boston, Massachusetts

**Keywords:** eosinophilic fasciitis, groove sign, monoclonal gammopathy, morphea, systemic sclerosis

*To the Editor:* We read with great interest, “Overlap of generalized morphea and eosinophilic fasciitis after recreational exposure to epoxy resin,” by Hewitt et al.^1^ In their article, the authors describe how it may be challenging to distinguish deep morphea from eosinophilic fasciitis (EF) and that these conditions are often thought of as existing along a spectrum. The authors list a symmetrical distribution, prominent inflammatory phase, and peripheral eosinophilia as clues that favor EF over morphea. In our experience, additional findings that favor EF include the groove sign, circumferential induration of the extremities (whereas morphea is characterized by discrete indurated plaques), and fascial involvement on magnetic resonance imaging. While it is true that many experts believe morphea and EF exist along a spectrum, attempting to differentiate between the 2 conditions is important as patients with EF, unlike morphea, may have an associated monoclonal gammopathy and should be screened accordingly. Furthermore, the authors highlight that their patient lacked dilated nailfold capillaries, an important finding that helps differentiate morphea/EF from systemic sclerosis (SSc). Other clues that favor morphea/EF over SSc include the absence of Raynaud’s phenomenon and lack of puffiness or induration of the distal digits. Distinguishing between morphea/EF and SSc is crucial; morphea/EF lacks the potential extracutaneous manifestations of SSc, and whereas systemic corticosteroids are first-line therapy for EF, they are typically avoided in SSc due to the risk of scleroderma renal crisis. We thank Hewitt et al for their contribution to the literature on these rare sclerosing conditions.

## Conflicts of interest

Dr Lo Sicco is a consultant for Pfizer and Aquis, has been an investigator for Regen Lab, is an investigator for Pfizer, and is a board member for the Scarring Alopecia Foundation. Dr LaChance is a principal investigator for Pfizer; co-investigator for Merck; consultant for Cowen, MedaCorp, Guidepoint, and Pfizer; and is on the advisory board for Johnson & Johnson. Dr Vleugels is a principal investigator or consultant for Pfizer, AstraZeneca, Apogee, Priovant, BMS, and Lilly. Dr Femia reports consulting fees from Octagon Therapeutics, Timber Pharmaceuticals, and Guidepoint. Dr Mazori has no conflicts of interest to disclose.
